# Preoperative Platelet-to-Lymphocyte Ratio as a Predictor of Recurrence and Recurrence-Free Survival in Non-Muscle-Invasive Bladder Cancer Across Different Intravesical Therapies

**DOI:** 10.3390/jcm15135199

**Published:** 2026-07-03

**Authors:** Muhammet İhsan Öztürk, Musa Ekici, Cemil Aydın, Mustafa Serdar Çağlayan, Mücahit Doğan, Mehmet Murat Baykam

**Affiliations:** 1 Alaca State Hospital, Çorum 19600, Turkey; 2 Department of Urology, Faculty of Medicine, Hitit University, Çorum 19040, Turkey; musaekici40@gmail.com (M.E.); cemilaydin78@yahoo.com.tr (C.A.); serdar.09.09@hotmail.com (M.S.Ç.); mbaykam@yahoo.com (M.M.B.); 3 Mengücek Gazi Training and Research Hospital, Erzincan 24100, Turkey; drmucahitdogan@outlook.com

**Keywords:** non-muscle invasive bladder cancer, platelet-to-lymphocyte ratio, recurrence, recurrence-free survival, Bacillus Calmette–Guérin, intravesical therapy, thermochemotherapy, inflammation markers, uro-oncology

## Abstract

**Background/Objectives:** Non-muscle invasive bladder cancer (NMIBC) is characterized by high recurrence rates despite appropriate treatment and surveillance. Identifying inexpensive and readily available biomarkers capable of improving risk stratification remains an important clinical challenge. The platelet-to-lymphocyte ratio (PLR), a marker of systemic inflammation, has emerged as a potential prognostic indicator in several malignancies. This study aimed to evaluate the association between preoperative PLR, tumor recurrence, and recurrence-free survival (RFS) in NMIBC patients treated with intravesical Bacillus Calmette–Guérin (BCG) or thermochemotherapy. **Methods:** This retrospective study included 153 patients diagnosed with NMIBC between January 2020 and January 2024. All patients underwent transurethral resection of bladder tumor (TURBT) followed by intravesical BCG (n = 123) or thermochemotherapy (n = 30). Preoperative PLR was calculated from complete blood counts obtained before surgery. Receiver operating characteristic (ROC) analysis was used to determine the optimal PLR cut-off value. Recurrence-free survival was evaluated using Kaplan–Meier survival analysis and Cox proportional hazards regression models. **Results:** During a mean follow-up period of approximately 19 months, recurrence was observed in 35.8% of patients treated with BCG and 30% of those treated with thermochemotherapy. ROC analysis demonstrated good discriminatory ability for recurrence prediction (AUC = 0.831, 95% CI: 0.761–0.901, *p* < 0.001) and identified an optimal PLR threshold of 120. Patients with elevated PLR values demonstrated higher recurrence rates and shorter recurrence-free survival. Kaplan–Meier analysis revealed a clear separation of survival curves according to PLR status. In multivariable Cox regression analysis, PLR > 120 remained independently associated with recurrence-free survival in the BCG group (HR = 2.703, 95% CI: 1.118–6.534, *p* = 0.027), whereas only a borderline association was observed in the thermochemotherapy group (HR = 23.265, 95% CI: 0.952–568.336, *p* = 0.054). **Conclusions:** Elevated preoperative PLR was associated with recurrence and recurrence-free survival in patients with NMIBC. The prognostic value of PLR appeared to be more pronounced in patients receiving intravesical BCG therapy. Given its low cost, accessibility, and ease of calculation, PLR may serve as a useful adjunctive biomarker for clinical risk stratification when used alongside established clinicopathological prognostic factors. Further prospective multicenter studies are required to validate these findings.

## 1. Introduction

Bladder cancer is one of the most frequently diagnosed malignancies of the urinary tract and remains a significant cause of cancer-related morbidity and mortality worldwide [1,2,3,4]. According to recent global cancer statistics, bladder cancer accounts for more than half a million new cases annually and represents a substantial healthcare burden because of its high recurrence rate and lifelong surveillance requirements [1]. Approximately 70–80% of newly diagnosed bladder cancers are classified as non-muscle-invasive bladder cancer (NMIBC), including Ta, T1, and carcinoma in situ (CIS) lesions [3,4]. Although patients with NMIBC generally experience more favorable survival outcomes than those with muscle-invasive disease, recurrence remains a major challenge in clinical practice, occurring in a substantial proportion of patients despite appropriate treatment and follow-up [3,4,5].

Current management of NMIBC relies on complete transurethral resection of the bladder tumor (TURBT) followed by risk-adapted intravesical therapy [3,4]. Intravesical Bacillus Calmette–Guérin (BCG) remains the standard adjuvant treatment for intermediate- and high-risk disease due to its proven efficacy in reducing recurrence and progression rates [3]. Nevertheless, recurrence continues to occur even among adequately treated patients, highlighting the biological heterogeneity of NMIBC. Furthermore, recurring global shortages of BCG have stimulated growing interest in alternative treatment modalities, including device-assisted intravesical therapies such as thermochemotherapy [6,7,8]. Consequently, identifying reliable biomarkers capable of improving risk stratification and guiding individualized treatment decisions has become increasingly important.

Several prognostic models have been developed to estimate recurrence and progression risks in NMIBC. Among these, the European Organization for Research and Treatment of Cancer (EORTC) risk tables remain one of the most widely utilized tools in daily clinical practice [5]. These models incorporate established clinicopathological variables, including tumor stage, grade, size, multiplicity, and prior recurrence history. Although such systems provide valuable prognostic information, their predictive accuracy remains limited because they do not fully account for biological processes underlying tumor behavior and host response [3,5]. Therefore, increasing attention has been directed toward easily accessible biomarkers that may complement existing risk assessment strategies.

Accumulating evidence indicates that systemic inflammation plays a pivotal role in cancer initiation, progression, angiogenesis, and immune evasion [9,10,11]. Chronic inflammatory responses may contribute to tumor development through the release of cytokines, growth factors, and other mediators that facilitate malignant transformation and disease progression [9]. Moreover, cancer-related inflammation has been recognized as one of the fundamental hallmarks of malignancy and is increasingly associated with adverse oncological outcomes across multiple tumor types [10,11]. Consequently, inflammation-based biomarkers derived from routine laboratory investigations have emerged as attractive prognostic tools in oncology.

Among these markers, the platelet-to-lymphocyte ratio (PLR) has gained considerable attention because of its simplicity, low cost, and widespread availability [12]. Platelets are known to promote tumor growth by supporting angiogenesis, enhancing tumor cell survival, facilitating metastatic dissemination, and protecting malignant cells from immune-mediated destruction [9,11]. In contrast, lymphocytes play a crucial role in antitumor immunity through cytotoxic activity and immune surveillance mechanisms [10]. Therefore, an elevated PLR may reflect both increased tumor-promoting inflammatory activity and reduced host immune competence, resulting in a biological environment that favors tumor recurrence and progression.

The prognostic significance of PLR has been investigated in numerous solid malignancies, including colorectal, gastric, lung, renal, and urothelial cancers [12,13,14,15]. Previous studies and meta-analyses have consistently demonstrated associations between elevated PLR and adverse oncological outcomes, including reduced survival and increased recurrence risk [12,13,14,15]. In bladder cancer specifically, several investigators have reported that elevated preoperative PLR is associated with poorer recurrence-free survival and unfavorable clinical outcomes following TURBT and intravesical treatment [13,16,17,18]. Moreover, recent studies evaluating systemic inflammatory markers in urothelial carcinoma have emphasized their potential role in risk stratification and personalized patient management [14,15,19,20].

Despite these promising findings, the available literature remains heterogeneous. While several studies have identified PLR as an independent predictor of recurrence and survival outcomes, others have reported weaker or non-significant associations after adjustment for conventional clinicopathological factors [17,18,20]. Differences in patient populations, treatment protocols, statistical methodologies, follow-up duration, and PLR cut-off values may partially explain these discrepancies. Furthermore, most published studies have focused primarily on patients receiving intravesical BCG therapy, whereas evidence regarding patients treated with thermochemotherapy remains relatively limited [6,7,8].

In addition to inflammatory biomarkers, other factors such as variant histology, smoking status, and tumor characteristics have been shown to influence recurrence risk and disease progression in NMIBC [21,22]. The integration of biological markers with established clinical predictors may therefore improve the accuracy of prognostic assessment and support more individualized treatment strategies.

Given the increasing interest in inflammation-based biomarkers and the expanding use of alternative intravesical therapies, further investigation of PLR in different treatment settings is warranted. Therefore, the aim of the present study was to evaluate the association between preoperative PLR and oncological outcomes in patients with NMIBC treated with either intravesical BCG or thermochemotherapy following TURBT. Specifically, we sought to investigate the relationship between preoperative PLR, tumor recurrence, and recurrence-free survival (RFS), and to assess the potential clinical utility of PLR as an adjunctive biomarker for risk stratification across different intravesical treatment modalities.

## 2. Materials and Methods

### 2.1. Study Design and Patient Population

This retrospective observational study was conducted at the Department of Urology of Hitit University Faculty of Medicine and affiliated institutions. Medical records of consecutive patients diagnosed with non-muscle invasive bladder cancer (NMIBC) between January 2020 and January 2024 were retrospectively reviewed. This study was conducted as part of the corresponding author’s postgraduate thesis. Ethical approval was obtained from the Non-Interventional Ethics Committee of Hitit University Faculty of Medicine on 21 August 2023, prior to the statistical analyses. The study retrospectively included eligible patients treated between January 2020 and January 2024. Statistical analyses were initiated in January 2024 using the retrospectively collected data, and the corresponding author’s thesis was completed and presented in February 2024. All patients underwent complete transurethral resection of bladder tumor (TURBT) performed by experienced urologists and subsequently received adjuvant intravesical treatment according to contemporary guideline recommendations and institutional practice patterns.

A total of 153 patients met the eligibility criteria and were included in the final analysis. Among them, 123 patients received intravesical Bacillus Calmette–Guérin (BCG) immunotherapy, whereas 30 patients underwent intravesical thermochemotherapy. Treatment selection was based on risk stratification, treatment availability, physician preference, and individual patient characteristics. Demographic, clinical, pathological, laboratory, treatment, and follow-up data were collected from institutional electronic medical records.

### 2.2. Inclusion and Exclusion Criteria

Patients were eligible for inclusion if they met all of the following criteria: (i) histologically confirmed NMIBC (Ta, T1, or carcinoma in situ); (ii) complete TURBT followed by intravesical treatment with either BCG or thermochemotherapy; and (iii) availability of complete clinical, pathological, laboratory, and follow-up data.

Patients were excluded if they had: (i) active infection at the time of diagnosis; (ii) known hematological disorders; (iii) previous systemic chemotherapy; (iv) concomitant inflammatory conditions that could significantly affect hematological parameters; or (v) incomplete clinical records. Patients who were switched to thermochemotherapy due to BCG intolerance after treatment initiation were also excluded from the analysis.

### 2.3. Data Collection and Clinical Variables

Baseline demographic and clinical variables included age, sex, smoking status, occupational exposure history, and comorbid diseases. Tumor-related variables included pathological stage, tumor grade, tumor size, tumor multiplicity, presence of concomitant carcinoma in situ (CIS), and variant histology.

Co-existing diseases were defined as the presence of one or more chronic medical conditions, including hypertension, diabetes mellitus, coronary artery disease, chronic obstructive pulmonary disease, or other clinically significant comorbidities documented in the medical records.

Occupational exposure was defined as a history of employment associated with exposure to recognized bladder carcinogens, including aromatic amines, industrial chemicals, dyes, rubber, leather, textile products, paint, petroleum-derived products, or related occupational hazards.

Laboratory parameters were obtained from peripheral venous blood samples collected within one week before TURBT. Complete blood count measurements were performed using standardized institutional laboratory protocols. Platelet counts and absolute lymphocyte counts were recorded for all patients. Additional laboratory and clinical information was reviewed to exclude patients with active infectious or hematological conditions that could influence inflammatory markers.

Histopathological specimens were evaluated by dedicated genitourinary pathologists according to the World Health Organization (WHO) classification system and TNM staging criteria.

### 2.4. Definition of Platelet-to-Lymphocyte Ratio

The platelet-to-lymphocyte ratio (PLR) was calculated by dividing the absolute platelet count by the absolute lymphocyte count obtained from preoperative complete blood count analyses. Receiver operating characteristic (ROC) curve analysis was performed to determine the optimal PLR cut-off value for predicting recurrence. The optimal threshold was identified using the maximum Youden index criterion. Sensitivity, specificity, and area under the curve (AUC) values were subsequently calculated.

### 2.5. Intravesical Treatment Protocols

Patients in the BCG group received standard intravesical BCG treatment consisting of a six-week induction course followed by maintenance therapy according to institutional protocols and contemporary guideline recommendations whenever clinically appropriate.

Patients in the thermochemotherapy group were treated using the Unithermia^®^ hyperthermic intravesical chemotherapy system. During each treatment session, epirubicin (50 mg/25 mL) was heated to approximately 42 °C and circulated intravesically for 50 min. Treatment consisted of eight weekly induction instillations followed by four monthly maintenance sessions. Treatment compliance and completion status were documented during follow-up.

### 2.6. Follow-Up and Outcome Assessment

Following completion of initial treatment, patients were followed according to standard NMIBC surveillance protocols. Follow-up evaluations included physical examination, urine cytology when clinically indicated, and office cystoscopy.

Surveillance cystoscopy was generally performed every three months during the first two years, every six months thereafter, and annually according to individual risk profiles and guideline recommendations. Additional diagnostic procedures were performed whenever recurrence was suspected based on clinical, cytological, or cystoscopic findings.

The primary outcome of the study was tumor recurrence. Recurrence was defined as histologically confirmed urothelial carcinoma detected during follow-up after complete TURBT and initiation of intravesical treatment.

Recurrence-free survival (RFS) was defined as the interval between the date of TURBT and the date of first documented recurrence. Patients without recurrence were censored at the date of their last available follow-up visit.

Secondary outcomes included progression to muscle-invasive bladder cancer, development of distant metastasis, and requirement for radical cystectomy.

### 2.7. Statistical Analysis

Statistical analyses were performed using SPSS software version 21.0 (IBM Corp., Armonk, NY, USA). Continuous variables were expressed as mean ± standard deviation or median (range), depending on data distribution, whereas categorical variables were presented as frequencies and percentages.

Normally distributed variables were reported as mean ± standard deviation, whereas non-normally distributed variables were expressed as median and range.

The normality of continuous variables was assessed using the Kolmogorov–Smirnov and Shapiro–Wilk tests. Comparisons between groups were performed using the Mann–Whitney U test for continuous variables and the chi-square test for categorical variables.

Receiver operating characteristic (ROC) curve analysis was performed to determine the optimal PLR threshold for predicting recurrence. Sensitivity, specificity, and area under the curve (AUC) values were calculated.

Kaplan–Meier survival analysis was used to estimate recurrence-free survival, and survival curves were compared using the log-rank test.

Univariable and multivariable Cox proportional hazards regression analyses were performed to identify factors associated with recurrence-free survival. Variables considered clinically relevant or statistically significant in univariable analyses were included in the multivariable model. Hazard ratios (HRs) and corresponding 95% confidence intervals (CIs) were calculated.

A two-sided *p*-value < 0.05 was considered statistically significant.

### 2.8. Ethical Approval

This study was approved by the Non-Interventional Ethics Committee of Hitit University Faculty of Medicine (approval date: 21 August 2023; decision no: 2023-86). The study was conducted in accordance with the principles of the Declaration of Helsinki. Due to the retrospective design of the study and the use of anonymized patient data, the requirement for informed consent was waived by the ethics committee.

### 2.9. Data Availability

The data supporting the findings of this study are available from the corresponding author upon reasonable request. Data sharing is subject to institutional regulations and patient confidentiality requirements.

### 2.10. Use of Generative Artificial Intelligence

The authors did not use generative artificial intelligence (GenAI) tools for data generation, data analysis, interpretation of results, or manuscript preparation.

## 3. Results

### 3.1. Patient Characteristics

A total of 153 patients with non-muscle-invasive bladder cancer (NMIBC) were included in the study, of whom 123 received intravesical Bacillus Calmette–Guérin (BCG) therapy and 30 underwent intravesical thermochemotherapy.

The mean age was 71.6 years in the BCG group and 67.3 years in the thermochemotherapy group. The majority of patients were male in both groups (95.1% and 93.3%, respectively). Smoking history was present in a substantial proportion of patients, and baseline demographic and clinical characteristics were comparable between groups.

Co-existing diseases were present in 78 patients (63.5%) in the BCG group and 21 patients (70.0%) in the thermochemotherapy group. Occupational exposure was documented in 7 patients (5.7%) receiving BCG therapy, whereas no patients in the thermochemotherapy group had a documented history of occupational exposure (Table 1).

### 3.2. Pathological Characteristics

Regarding pathological characteristics, tumor stage in the BCG group was classified as pTa in 39 patients (31.7%), pT1 in 76 patients (61.8%), and concomitant carcinoma in situ (CIS) was present in 19 patients (15.4%). Tumors were classified as low-grade in 39 patients (31.7%) and high-grade in 76 patients (61.8%). The mean tumor size was 3.3 ± 1.9 cm, and the mean tumor number was 1.57. Variant histology was identified in 7 patients (5.7%), consisting of nested and squamous differentiation variants.

In the thermochemotherapy group, tumor stage was classified as pTa in 10 patients (33.3%) and pT1 in 20 patients (66.7%). Low-grade tumors were observed in 17 patients (56.7%), whereas high-grade tumors were present in 13 patients (43.3%). The mean tumor size was 3.4 ± 1.2 cm, and the mean tumor number was 1.70. No cases of concomitant CIS or variant histology were identified in the thermochemotherapy group (Table 2).

### 3.3. Recurrence Outcomes

During a mean follow-up period of approximately 19 months, tumor recurrence was observed in 35.8% of patients in the BCG group and 30% of patients in the thermochemotherapy group.

The mean preoperative platelet-to-lymphocyte ratio (PLR) was 120.2 ± 32.0 in the BCG group and 145.1 ± 77.2 in the thermochemotherapy group. Tumor recurrence occurred in 44 patients (35.8%) receiving BCG therapy and in 9 patients (30.0%) treated with thermochemotherapy. Higher PLR values were generally associated with increased recurrence rates in both treatment groups (Table 3).

### 3.4. ROC Analysis

**Figure 1:**
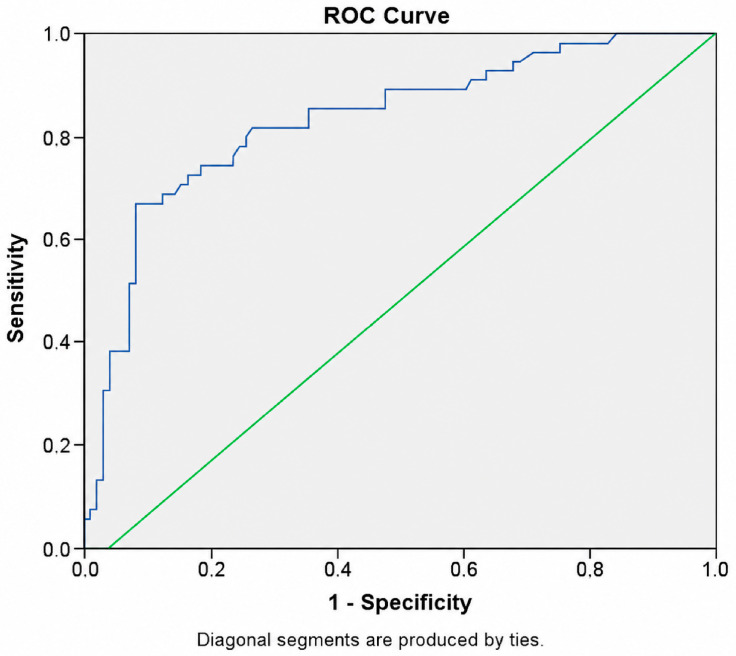
ROC curve showing the relationship between PLR and recurrence.

Receiver operating characteristic (ROC) curve analysis was performed to evaluate the ability of preoperative PLR to predict tumor recurrence. The analysis demonstrated good discriminatory performance, with an area under the curve (AUC) of 0.831 (95% CI: 0.761–0.901, *p* < 0.001) (Figure 1).

The optimal PLR cut-off value for predicting recurrence was identified as 120. At this threshold, the sensitivity and specificity were 77.4% and 76.0%, respectively. Based on this cut-off value, patients were subsequently stratified into two groups (PLR < 120 and PLR ≥ 120) for survival analyses.

### 3.5. Kaplan–Meier Survival Analysis

Kaplan–Meier survival analysis demonstrated that patients with PLR ≥ 120 had shorter recurrence-free survival (RFS) compared to those with PLR < 120.

**Figure 2:**
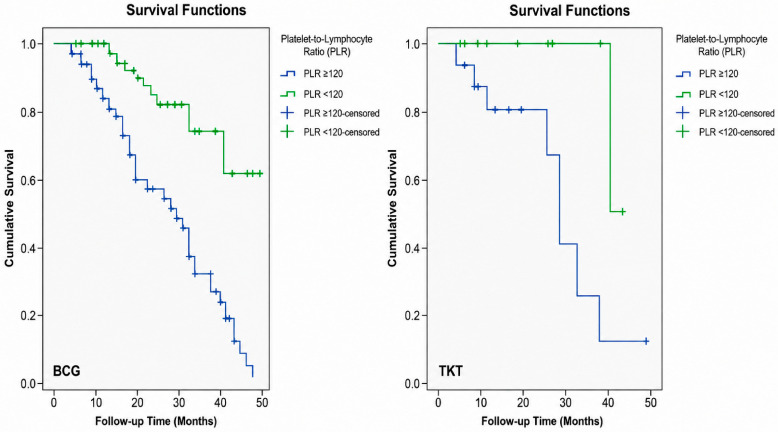
Recurrence-free survival curve for PLR 120 threshold in BCG and TKT treated patients.

The median RFS for the overall cohort was 25 months (95% CI: 16.2–33.8), and the mean RFS was 26.7 months. A visible separation between survival curves was observed between the PLR groups (Figure 2).

### 3.6. Cox Proportional Hazards Regression Analysis

Multivariable Cox proportional hazards regression analyses were performed to identify factors associated with recurrence-free survival in both treatment groups. In the thermochemotherapy group, PLR > 120 demonstrated a strong but borderline association with recurrence-free survival (HR = 23.265, 95% CI: 0.952–568.336, *p* = 0.054); however, this association did not reach statistical significance after adjustment for age, tumor size, and tumor number. Likewise, age (HR = 1.068, 95% CI: 0.980–1.163, *p* = 0.134), tumor size (HR = 1.424, 95% CI: 0.637–3.180, *p* = 0.389), tumor number (HR = 2.122, 95% CI: 0.797–5.651, *p* = 0.132), and continuous PLR values (HR = 0.995, 95% CI: 0.985–1.006, *p* = 0.366) were not independently associated with recurrence-free survival. In the BCG group, PLR > 120 remained independently associated with recurrence-free survival in the multivariable model (HR = 2.703, 95% CI: 1.118–6.534, *p* = 0.027). Patients with elevated PLR demonstrated a 2.7-fold higher risk of recurrence compared with those with PLR < 120. In contrast, age (HR = 0.987, 95% CI: 0.951–1.024, *p* = 0.491), tumor size (HR = 1.094, 95% CI: 0.885–1.352, *p* = 0.405), tumor number (HR = 1.007, 95% CI: 0.714–1.421, *p* = 0.966), and continuous PLR values (HR = 1.008, 95% CI: 0.997–1.020, *p* = 0.146) did not demonstrate statistically significant associations with recurrence-free survival. The results of the multivariable Cox proportional hazards regression analysis for recurrence-free survival in the BCG group are summarized in Table 4.

The corresponding multivariable Cox proportional hazards regression analysis for the thermochemotherapy group is presented in Table 5.

### 3.7. Subgroup Analysis

Subgroup analyses demonstrated that elevated PLR (≥120) was associated with recurrence in both treatment groups.

In the BCG group, recurrence appeared to be more frequent among patients with elevated PLR, smoking history, and variant histology. In the thermochemotherapy group, recurrence was associated with elevated PLR and higher tumor stage (T1). These findings should be interpreted cautiously because of the relatively small sample size of the thermochemotherapy cohort.

## 4. Discussion

The present study evaluated the association between preoperative platelet-to-lymphocyte ratio (PLR) and oncological outcomes in patients with non-muscle invasive bladder cancer (NMIBC) treated with intravesical Bacillus Calmette–Guérin (BCG) or thermochemotherapy. Our findings demonstrated that elevated preoperative PLR was associated with increased recurrence rates and shorter recurrence-free survival in patients with NMIBC. Kaplan–Meier analyses showed a clear separation between survival curves according to PLR status, supporting the potential prognostic relevance of systemic inflammatory status in this patient population. These results support the growing body of evidence suggesting that systemic inflammatory markers may provide clinically relevant prognostic information in NMIBC and may contribute to improved risk stratification when used alongside established clinicopathological factors [13,14,15,16,17,18,19,20].

Although NMIBC is generally associated with favorable long-term cancer-specific survival, recurrence remains one of the greatest challenges in clinical management. Despite advances in endoscopic techniques, intravesical therapies, and surveillance protocols, recurrence continues to occur in a substantial proportion of patients during follow-up [3,4,5]. Consequently, there has been increasing interest in identifying biomarkers capable of improving current prognostic models and enabling more individualized patient management strategies. In this context, inflammation-based hematological parameters have emerged as attractive candidates because they are inexpensive, readily available, and routinely measured in clinical practice [14,15,19].

The biological relationship between systemic inflammation and cancer progression has been increasingly recognized over the past two decades. Chronic inflammation contributes to multiple stages of carcinogenesis, including cellular proliferation, angiogenesis, immune evasion, tumor invasion, and metastatic dissemination [9,10,11]. Cancer-related inflammatory responses may alter the tumor microenvironment through the release of cytokines, chemokines, and growth factors that facilitate malignant progression [9,10]. Consequently, systemic inflammatory biomarkers have been extensively investigated as potential prognostic indicators across numerous malignancies.

PLR represents one of the most widely studied inflammation-based biomarkers. Elevated platelet counts may reflect tumor-driven inflammatory activity and increased production of proangiogenic mediators such as vascular endothelial growth factor and platelet-derived growth factor [9,11]. Platelets may also facilitate tumor cell survival within the circulation and protect malignant cells from immune-mediated destruction. In contrast, lymphocytes constitute a fundamental component of antitumor immunity and play a central role in immune surveillance mechanisms [10]. Therefore, an elevated PLR may indicate both increased tumor-promoting inflammatory activity and impaired host immune response, providing a biologically plausible explanation for its association with unfavorable oncological outcomes [7,12].

Our findings are broadly consistent with previous investigations evaluating the prognostic significance of PLR in bladder cancer. Wang et al. reported that elevated PLR was associated with poorer oncological outcomes in bladder cancer patients, while subsequent studies demonstrated significant relationships between increased PLR and recurrence following transurethral resection [13,16]. More recently, Wu et al. observed that elevated PLR was associated with adverse outcomes in NMIBC patients receiving intravesical BCG therapy, supporting the potential clinical value of inflammation-based biomarkers in this setting [17]. Similarly, recent systematic reviews and meta-analyses have concluded that elevated PLR is associated with increased recurrence risk and less favorable survival outcomes in urothelial malignancies [18,20].

The ROC analysis further supported the prognostic relevance of PLR, demonstrating good discriminatory performance for recurrence prediction with an AUC of 0.831. This finding suggests that PLR may have practical value as a simple and readily available marker for identifying patients at increased risk of recurrence.

An important observation of the present study was the difference observed between treatment modalities in multivariable analyses. In patients receiving intravesical BCG therapy, PLR > 120 remained independently associated with recurrence-free survival after adjustment for age, tumor size, and tumor number (HR = 2.703, 95% CI: 1.118–6.534, *p* = 0.027). In contrast, only a borderline association was observed in the thermochemotherapy group. These findings suggest that the prognostic significance of PLR may be more pronounced among patients treated with BCG. However, given the relatively small number of patients in the thermochemotherapy cohort, these results should be interpreted cautiously and require validation in larger prospective studies.

An additional consideration is the biological and clinical heterogeneity of NMIBC. Established prognostic factors, including tumor stage, grade, concomitant CIS, tumor multiplicity, and variant histology, may influence both recurrence risk and the performance of inflammation-based biomarkers. Therefore, the prognostic value of PLR may not be uniform across all NMIBC risk categories. Although PLR demonstrated significant prognostic value in the present cohort, particularly among patients receiving BCG therapy, its predictive performance may vary according to individual clinicopathological characteristics. Future prospective studies with larger cohorts and detailed subgroup analyses are warranted to determine whether PLR performs differently across specific NMIBC risk groups.

The increasing utilization of thermochemotherapy further highlights the clinical relevance of our findings. Device-assisted intravesical therapies have gained attention as potential alternatives or complements to conventional BCG treatment, particularly in the setting of recurrent disease and global BCG shortages [6]. Hyperthermic intravesical chemotherapy has demonstrated encouraging oncological outcomes in selected patient populations by enhancing drug penetration and cytotoxic activity within the bladder wall [7,8]. However, validated biomarkers capable of predicting treatment response and recurrence risk in these patients remain scarce. Therefore, our findings may provide additional evidence supporting the potential role of PLR in contemporary NMIBC management irrespective of the chosen intravesical treatment modality.

In addition to PLR, several clinicopathological variables demonstrated associations with recurrence in subgroup analyses. Smoking status, tumor stage, and variant histology were among the factors associated with adverse outcomes. These findings are consistent with established prognostic models and previous literature evaluating NMIBC recurrence and progression [5,21,22]. Smoking remains one of the most important modifiable risk factors in bladder cancer and has been associated with increased recurrence and progression rates even after successful initial treatment [22]. Similarly, variant histology and higher pathological stage have long been recognized as indicators of more aggressive tumor biology and less favorable oncological outcomes [21].

From a clinical perspective, PLR offers several practical advantages. Unlike molecular biomarkers that often require specialized laboratory infrastructure and increased costs, PLR can be calculated rapidly from routine complete blood count analyses without additional testing [12]. This makes PLR particularly attractive for everyday clinical practice and potentially useful in healthcare systems with limited access to advanced molecular diagnostics. Furthermore, incorporation of PLR into existing prognostic models may improve identification of patients who require closer surveillance, more intensive intravesical treatment, or earlier consideration of alternative therapeutic strategies.

The increasing emphasis on personalized medicine further strengthens interest in inflammation-based biomarkers. Contemporary management of NMIBC seeks to optimize oncological outcomes while minimizing overtreatment and unnecessary healthcare expenditures. Biomarkers capable of identifying patients at increased risk of recurrence may facilitate more individualized surveillance schedules and treatment decisions. In recent years, several authors have suggested that inflammation-based biomarkers could be incorporated into predictive nomograms and risk calculators to enhance prognostic accuracy [14,15,23]. Although PLR alone is unlikely to replace established clinicopathological factors, it may provide additional biological information that complements traditional risk assessment approaches.

Several limitations of the present study should be acknowledged. First, the retrospective design introduces an inherent risk of selection bias and limits the ability to establish causal relationships. Second, the study was conducted at a single institution, potentially affecting the generalizability of the findings. Third, the relatively small number of patients in the thermochemotherapy group may have limited statistical power and subgroup analyses. Fourth, inflammatory markers may be influenced by conditions unrelated to cancer, including occult infections, chronic inflammatory diseases, medications, and comorbidities. Although major confounding factors were considered during patient selection, residual confounding cannot be completely excluded.

Additional methodological considerations should also be recognized. No universally accepted PLR threshold currently exists, and cut-off values vary considerably across published studies [13,17,18]. Differences in patient characteristics, laboratory methods, follow-up duration, and statistical approaches may contribute to this variability. Therefore, caution is warranted when comparing PLR thresholds between different investigations or applying them directly to external populations.

An additional limitation relates to the relatively small sample size of the thermochemotherapy group, which may have reduced statistical power and contributed to the borderline significance observed in multivariable analyses. Therefore, the absence of an independent association in this subgroup should not be interpreted as definitive evidence of a lack of prognostic value.

Despite these limitations, the present study contributes additional evidence supporting the prognostic relevance of systemic inflammatory biomarkers in NMIBC. The inclusion of patients treated with both BCG and thermochemotherapy represents a notable strength and provides insight into the potential applicability of PLR across different intravesical treatment settings. Moreover, the use of routinely available laboratory data enhances the practical relevance and potential clinical utility of our findings.

Future prospective multicenter studies involving larger patient populations are required to validate these observations. Standardization of PLR cut-off values and statistical methodologies would improve comparability across studies and facilitate clinical implementation. Furthermore, combining PLR with molecular biomarkers, genomic signatures, and artificial intelligence-based prediction models may further improve recurrence prediction and support individualized treatment strategies in patients with NMIBC [14,15,23].

Overall, our findings support the concept that systemic inflammatory status is associated with recurrence risk in NMIBC. Elevated preoperative PLR was associated with recurrence and shorter recurrence-free survival, and PLR > 120 remained independently associated with recurrence-free survival in patients receiving intravesical BCG therapy. Given its low cost, widespread availability, and ease of calculation, PLR may represent a useful adjunctive biomarker for risk stratification in selected NMIBC populations. Nevertheless, larger prospective multicenter studies are required to validate these findings and further define the role of PLR across different NMIBC risk categories and treatment modalities.

## 5. Conclusions

In this study, elevated preoperative PLR was associated with recurrence and recurrence-free survival in patients with NMIBC. Independent prognostic significance was observed in the BCG subgroup, whereas a borderline association was observed in patients treated with thermochemotherapy. Patients with elevated PLR values experienced higher recurrence rates and shorter recurrence-free survival, suggesting that systemic inflammatory status may influence oncological outcomes following transurethral resection and adjuvant intravesical treatment.

Given its low cost, widespread availability, and ease of calculation from routine complete blood count measurements, PLR may represent a practical adjunctive biomarker for clinical risk assessment in NMIBC. Although PLR should not be considered a replacement for established clinicopathological prognostic factors, its integration into existing risk stratification models may improve the identification of patients at increased risk of recurrence and support more individualized surveillance and treatment strategies.

Further prospective multicenter studies involving larger patient populations, longer follow-up periods, and standardized PLR cut-off values are warranted to validate these findings and clarify the role of PLR within contemporary personalized management approaches for NMIBC.

## Figures and Tables

**Table 1:** Demographic and Clinical Characteristics of the Patients.

Variables	BCG n (%)	TKT n (%)
Age	71.6	67.3
Gender		
Female	6 (4.9)	2 (6.7)
Male	117 (95.1)	28 (93.3)
Smoking		
Smoker	33 (26.8)	7 (23.3)
Quit Smoking	81 (65.9)	16 (53.4)
Never Smoked	9 (7.3)	7 (23.3)
Occupational Exposure		
Present	7 (5.7)	0
Absent	116 (94.3)	30 (100)
Co-existing Diseases		
Present	78 (63.5)	21 (70)
Absent	45 (36.5)	9 (30)

**Table 2:** Baseline Tumor and Pathological Characteristics of the Study Population.

Variables	BCG n (%)	TKT n (%)
Mean Tumor Size (cm)	3.3	3.4
Mean Tumor Number	1.57	1.70
Pathological Stage		
Ta	39 (31.7)	10 (33.3)
T1	76 (61.8)	20 (66.7)
Tumor Grade		
Low Grade	39 (31.7)	17 (56.7)
High Grade	76 (61.8)	13 (43.3)
Concomitant CIS	19 (15.4)	0
Variant Histology	7 (5.7)	0

**Table 3:** Recurrence Outcomes and Preoperative PLR Value.

Variables	BCG n (%)	TKT n (%)
Platelet-Lymphocyte Ratio	120.2 ± 32 (67.4–273)	145.1 ± 77.2 (67.8–429.4)
Recurrence		
None	79 (64.2)	21 (70)
Present	44 (35.8)	9 (30)

**Table 4:** Multivariable Cox Proportional Hazards Regression Analysis of Factors Associated with Recurrence-Free Survival (RFS) in the BCG Group.

Variables	HR (Exp(B))	95% CI	*p*-Value
Age	0.987	0.951–1.024	0.491
Tumor Size	1.094	0.885–1.352	0.405
Tumor Number	1.007	0.714–1.421	0.966
PLR > 120 vs. ≤120	2.703	1.118–6.534	0.027

Abbreviations: HR, hazard ratio; CI, confidence interval; PLR, platelet-to-lymphocyte ratio; RFS, recurrence-free survival.

**Table 5:** Multivariable Cox Proportional Hazards Regression Analysis of Factors Associated with Recurrence-Free Survival (RFS) in the Thermochemotherapy Group.

Variables	HR (Exp(B))	95% CI	*p*-Value
Age	1.068	0.980–1.163	0.134
Tumor Size	1.424	0.637–3.180	0.389
Tumor Number	2.122	0.797–5.651	0.132
PLR > 120 vs. ≤120	23.265	0.952–568.336	0.054

Abbreviations: HR, hazard ratio; CI, confidence interval; PLR, platelet-to-lymphocyte ratio; RFS, recurrence-free survival.

## Data Availability

The data presented in this study are available from the corresponding author upon reasonable request. The data are not publicly available due to privacy and ethical restrictions.

## Abbreviations

The following abbreviations are used in this manuscript:

NMIBC	Non-muscle invasive bladder cancer
PLR	Platelet-to-lymphocyte ratio
RFS	Recurrence-free survival
BCG	Bacillus Calmette–Guérin
TURBT	Transurethral resection of bladder tumor
CIS	Carcinoma in situ
NLR	Neutrophil-to-lymphocyte ratio
TKT	Thermochemotherapy
